# Morphological characterization and interspecific variation among five species of *Ziziphus* genus to select superiors in Iran

**DOI:** 10.1186/s12870-023-04566-4

**Published:** 2023-11-08

**Authors:** Ali Khadivi

**Affiliations:** Department of Horticultural Sciences, Faculty of Agriculture and Natural Resources, Arak University, Arak, 38156-8-8349 Iran

**Keywords:** Breeding, *Ziziphus*, Phenotypic variation, Correlation, MRA

## Abstract

**Background:**

Several species of the genus *Ziziphus* are used worldwide for their medicinal and therapeutic properties. The present study aimed to investigate the phenotypic variation of five species of the *Ziziphus* genus, including *Z. jujuba* Mill. (25 accessions), *Z. mauritiana* Lam. (25 accessions), *Z. spina-christi* L. (25 accessions), *Z. nummularia* L. (10 accessions), and *Z. xylopyrus* Willd. (10 accessions) from Markazi, Sistan-va-Baluchestan, and Khuzestan provinces, Iran.

**Results:**

The investigated accessions have significant differences in terms of all the measured as revealed using analysis of variance (ANOVA, *P* < 0.01). The range of fruit weight was 0.43–1.29 g in *Z. jujuba*, 17.85–29.87 g in *Z. mauritiana*, 0.94–3.44 g in *Z. spina-christi*, 0.93–2.02 g in *Z. nummularia*, and 0.91–3.02 g in *Z. xylopyrus*. All the measured traits showed significant and positive correlations with each other. Multiple regression analysis (MRA) results showed that fruit length, stone width, stone weight, stone length, and fruit width have significant effects on fruit weight, and thus their fluctuations have a significant effect on increasing or decreasing fruit weight. The accessions were grouped into two main clusters using hierarchical cluster analysis. The first cluster (I) included all the accessions of *Z. mauritiana*, while the second cluster (II) contained the accessions of the rest species forming two sub-clusters.

**Conclusion:**

Based on the commercial characters, accessions no. 12, 13, 17, 23, and 24 in *Z. jujuba*, accessions no. 3, 9, 17, 18, 20, 22, and 23 in *Z. mauritiana*, accessions no. 5, 6, 8, 13, 19, 22, and 24 in *Z. spina-christi*, accessions no. 3, 7, and 9 in *Z. nummularia*, and accessions no. 2, 4, 7, and 10 in *Z. oxyphylla* showed the highest fruit weight and thus can be suggested as superior for cultivation or use in breeding programs due to having larger fruits.

## Introduction

Several species of the genus *Ziziphus* are used worldwide for their medicinal and therapeutic properties. In India, China, South America, South Africa, and the Middle East, some species of the *Ziziphus* genus are applied to treat diseases. Five species of the genus *Ziziphus*, including *Z. jujuba* Mill., *Z. nummularia* L., *Z. mauritiana* Lam., *Z. xylopyrus* Willd., and *Z. spina-christi* L. are distributed in most regions of the world, including Iran [1].

*Z. jujuba* (jujube) is a thorny tree that is resistant to cold and heat. The adaptability of this tree against harsh climatic conditions and soil type has caused its cultivation to develop in cold regions. *Z. jujuba* grows on poor-quality land and produces a relatively satisfactory result. *Z. jujuba* tree grows better in hot and dry climates, but it can also tolerate low winter temperatures of -29 ˚C [2]. *Z. jujuba* has different biological activities and has higher medicinal and nutritional value [3]. Since ancient times, in traditional Chinese medicine, dried fruits of this species have been used to treat tumors [4]. Also, in traditional Iranian medicine, the fruit of this species is used to eliminate cough and reduce blood pressure [2].

*Z. mauritiana* (Indian jujube) originated from tropical regions in South and East Asia and its height may reach 15 m. Its fruit is mostly available fresh in the market. *Z. mauritiana* needs a lot of light and low humidity, and its heat requirement is high. It also needs loamy to loamy and deep sandy soils with neutral to slightly alkaline pH. *Z. mauritiana* is traditionally used to treat various diseases. Its fruit extract is used for skin health [5] and also to reduce sunburn [6].

*Z. spina-christi* is a thorny shrub or a tree of medium height that shows high resistance to drought stress. *Z. spina-christi* has a long history in Arab traditional medicine and the consumption of its fruits helps to reduce lung problems. The most suitable temperature for the growth of *Z. spina-christi* is between 25 and 35 ˚C, while fruit formation decreases at temperatures higher than 35 ˚C. Temperatures below zero and freezing temperatures cause damage to young branches and developing fruits and cause a significant reduction in yield and tree growth. *Z. spina-christi* trees can easily withstand heavy winds and in most cases, they are used as windbreaks. Due to strong resistance to water shortage and having strong and deep roots, the *Z. spina-christi* tree needs very little water after the establishment stage and can continue life and produce crops even without water [7].

*Z. nummularia* is native to India, Pakistan, Afghanistan, Iran, Lebanon, and Zimbabwe. It is tolerant of a range of habitats, including hillsides, plains, ravines, cultivated areas, and dunes. The leaves are rounded like those of *Z. jujuba* but differ from those in having a pubescence on the adaxial surface. The plant is commonly found in arid areas, hills, plains, and agricultural fields. The fruits of *Z. nummularia* are used to fight colds [8] and its seeds are used to treat eye diseases [9].

*Z. xylopyrus* is a perennial shrub with immense medicinal potential and is dispersed all over Pakistan, China, Iran, and India. It is very common in foothill scrub jungles, up to 1200 m altitude. Fresh fruits of *Z. xylopyrus* are used to treat urinary problems, and pigments extracted from its fruit are used for leather production [10].

The basis of the breeding program of plants is genetic variation. Genetic diversity of plant species should be investigated for use in the management, conservation, breeding, and creation of living vegetation [11, 12]. Genetic diversity is investigated using several methods, among which morphological characterization is the most powerful method to determine the classification of plants and agricultural benefits [13]. Determining the genetic diversity in plant materials is of great importance and is the first and fundamental step to identifying, preserving, and maintaining the genetic resources, which are considered the basis for genetic research and breeding programs. The genetic diversity of domesticated plants has been stabilized due to the use of limited genetic bases in breeding programs, and the diversity of native cultivars is also decreasing [13]. Morphological classification is a useful guide to identifying species relationships and increases the knowledge of plant breeders and gene bank managers. Also, knowledge of the relationships between traits (regression and correlation relationships) can be useful for the development of new commercial cultivars and resistant and short bases. Morphological characterization of plants is one of the first steps to identify genetic resources [12].

In Iran, the genotypes of the genus *Ziziphus* are among the scattered trees that have rarely been cultivated in a uniform and commercial manner, and as a result, the identification of their beneficial effects has not received much attention from researchers. However, attention to the cultivation and processing of these plants will obtain high income and many jobs. Therefore, the present study aimed to investigate the phenotypic characterization and interspecific variation among five species of the *Ziziphus* genus to select the superiors.

## Materials and methods

### Plant material

**Fig. 1:**
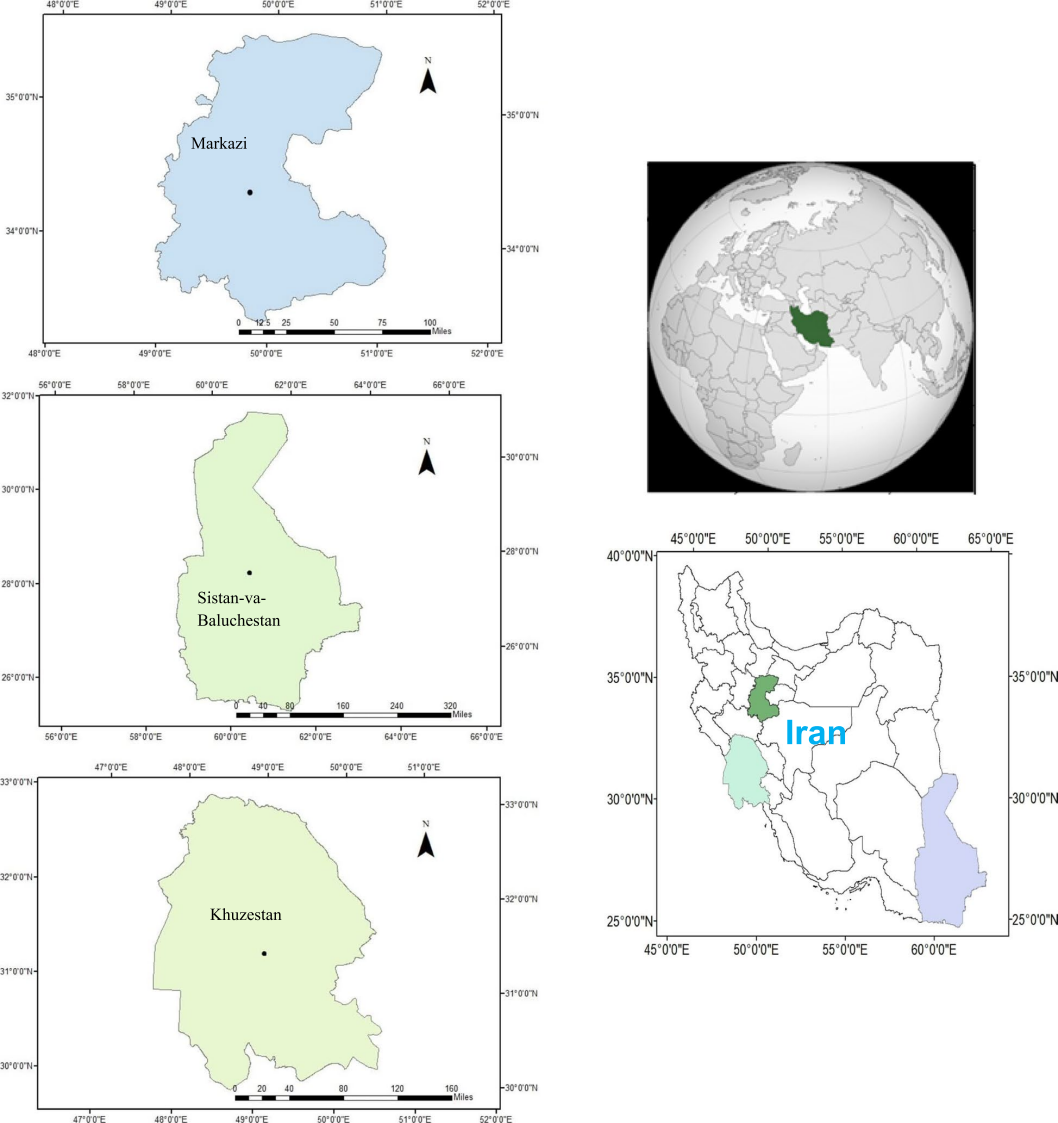
Geographic locations of collection sites of the studied five species of the *Ziziphus* genus in Iran

In total, 95 accessions belonging to five species of the *Ziziphus* genus, including *Z. jujuba* (25 accessions), *Z. mauritiana* (25 accessions), *Z. spina-christi* (25 accessions), *Z. nummularia* (10 accessions), and *Z. xylopyrus* (10 accessions) were studied from Markazi, Sistan-va-Baluchestan, and Khuzestan provinces, Iran (Fig. 1) for two consecutive years to determine morphological variation and also to select the promising accessions based on the quality of fruit. The identification of the species was performed by Prof. Dr. Ali Khadivi. A herbarium voucher specimen with sediment number ZZ-3457 was donated to a public available herbarium of the Faculty of Agriculture and Natural Resources of Arak University, Iran.

### The characteristics evaluated

To investigate the phenotypic variation between accessions and species, nine morphological traits were recorded. The 50 fruits and 50 leaves of each accession were measured and then their average was calculated. A digital caliper was used to measure quantitative traits related to the length and width of leaf, petiole, fruit, and stone, while a sensitive scale was used to measure fruit and stone weight.

### Statistical analysis

Analysis of variance (ANOVA) was done using SAS software [14]. Descriptive statistics, simple correlation between traits, and principal component analysis (PCA) were performed using SPSS (Version 16.0) software (SPSS Inc., Chicago, IL, USA) [15]. The coefficient of variation (CV) was calculated by dividing the standard deviation of each trait by the mean of that trait. In addition, SPSS software was used for multiple regression analysis (MRA) using the stepwise linear method, the purpose of which was to determine the independent traits affecting fruit weight. In MRA, *r*^2^ and *β* coefficients were calculated using regression analysis and were investigated for different traits related to traits. The *r*^2^ coefficient represents the multiple correlation coefficient and measures the correlation between fruit traits. Also, *β* is the standardized regression coefficient, which is calculated by MRA for each trait-related trait. Ward’s method and Euclidean distance coefficient using PAST software were applied to perform cluster analysis [16]. Also, a bi-plot was created using the first two main components (PC1/PC2) using PAST software.

## Results and discussion

The investigated accessions have significant differences in terms of all the measured traits as revealed using analysis of variance (ANOVA, *P* < 0.01). This result indicates the existence of phenotypic variation in the examined traits. In this case, selection can be made from the examined accessions.

 In *Z. jujuba*, CV ranged from 9.00 (in stone width) to 31.55% (in fruit weight). Also, the range of studied traits in this species was as follows: leaf length as 32.85–58.78 mm, leaf width as 11.57–28.68 mm, petiole length as 2.13–6.35 mm, fruit length as 9.51–16.52 mm, fruit width as 9.20-23.26 mm, fruit weight as 0.43–1.29 g, stone length as 6.76–12.15 mm, stone width as 5.50–7.77 mm, and stone weight as 0.08–0.25 g (Table 1). Khadivi et al. [17] reported a range of 26.33–84.05 mm for leaf length, 0.36–3.83 g for fruit weight, and 0.04–0.53 g for stone weight in *Z. jujuba.* Khadivi and Beigi [18] reported a range of 36.44–54.43 mm for leaf length, 2.72–6.42 g for fruit weight, and 0.31–0.47 g for stone weight in *Z. jujuba.* The wide range obtained for leaf and fruit-related characters in the present research was different from previous studies due to differences in the number of samples, environmental conditions, and genetic aspects.

**Table 1:** Statistical descriptive parameters for morphological traits used to study accessions belonging to five species of the *Ziziphus* genus

			*Z. jujuba*					*Z. mauritiana*					*Z. spina-christi*					*Z. nummularia*					*Z. xylopyrus*				
No.	Character	Unit	Min	Max	Mean	SD	CV (%)	Min	Max	Mean	SD	CV (%)	Min	Max	Mean	SD	CV (%)	Min	Max	Mean	SD	CV (%)	Min	Max	Mean	SD	CV (%)
1	Leaf length	mm	32.85	58.78	43.86	8.00	18.25	58.86	95.32	73.92	10.76	14.55	23.45	44.48	34.58	6.00	17.35	16.45	34.40	23.29	5.18	22.22	23.02	34.40	29.14	3.47	11.91
2	Leaf width	mm	11.57	28.68	19.19	3.82	19.91	35.08	75.83	49.16	10.90	22.17	17.37	30.38	23.94	3.84	16.05	9.80	23.70	15.38	3.93	25.58	17.02	23.00	20.38	1.75	8.58
3	Petiole length	mm	2.13	6.35	4.13	1.02	24.69	11.65	28.69	18.39	4.30	23.41	2.82	10.95	6.63	1.90	28.72	2.45	6.50	3.68	1.32	35.91	4.10	7.00	5.80	0.81	13.94
4	Fruit length	mm	9.51	16.52	12.28	1.83	14.86	31.21	45.33	38.40	4.24	11.05	11.16	17.72	13.89	1.75	12.60	10.32	15.40	12.70	1.70	13.42	11.45	16.33	13.10	1.57	11.96
5	Fruit width	mm	9.20	23.26	12.10	2.73	22.57	26.45	39.61	33.63	3.26	9.71	11.88	18.43	15.06	1.97	13.09	12.22	16.30	13.74	1.36	9.90	12.22	18.65	13.94	1.91	13.67
6	Fruit weight	g	0.43	1.29	0.81	0.26	31.55	17.85	29.87	23.58	4.62	19.61	0.94	3.44	2.10	0.80	37.88	0.93	2.02	1.44	0.36	24.90	0.91	3.02	1.51	0.62	41.02
7	Stone length	mm	6.76	12.15	9.25	1.42	15.38	16.75	24.65	21.15	2.55	12.07	7.98	12.13	9.71	1.10	11.36	7.32	10.80	9.00	1.19	13.27	7.33	10.70	9.05	1.03	11.36
8	Stone width	mm	5.50	7.77	6.23	0.56	9.00	8.02	12.58	10.19	1.32	13.00	6.93	10.02	8.50	0.89	10.41	7.30	9.62	8.22	0.99	12.09	7.02	9.44	8.16	0.84	10.34
9	Stone weight	g	0.08	0.25	0.16	0.04	25.47	0.90	2.38	1.60	0.57	35.86	0.25	0.79	0.47	0.15	32.33	0.31	0.81	0.60	0.21	35.27	0.35	0.82	0.59	0.19	31.55

In *Z. mauritiana*, CV ranged from 9.71 (in fruit width) to 35.86% (in stone weight). Also, a range of studied traits in this species was as follows: leaf length as 58.86–95.32 mm, leaf width as 35.08–75.83 mm, petiole length as 11.65–28.69 mm, fruit length as 31.21–45.33 mm, fruit width as 26.45–39.61 mm, fruit weight as 17.85–29.87 g, stone length as 16.75–24.65 mm, stone width as 8.02–12.58 mm, and stone weight as 0.90–2.38 g (Table 1). Mirheidari et al. [19] reported a range of 57.98–96.35 mm for leaf length, 15.68–33.62 g for fruit weight, and 0.76–2.52 g for stone weight in *Z. mauritiana.* The wide range obtained for leaf and fruit-related characters in the present research was near to the findings of Mirheidari et al. [19] due to the same environmental conditions.

In *Z. spina-christi*, CV ranged from 10.41 (in stone width) to 37.88% (in fruit weight). Also, the range of studied traits in this species was as follows: leaf length as 23.45–44.48 mm, leaf width as 17.37–30.38 mm, petiole length as 2.82–10.95 mm, fruit length as 11.16–17.72 mm, fruit width as 11.88–18.43 mm, fruit weight as 0.94–3.44 g, stone length as 7.98–12.13 mm, stone width as 6.93–10.02 mm, and stone weight as 0.25–0.79 g (Table 1). Norouzi et al. [20] reported a range of 18.90–37.00 mm for leaf length, 1.10–3.08 g for fruit weight, and 0.32–1.17 g for stone weight in *Z. spina-christi*. Zandiehvakili and Khadivi [21] reported a range of 23.68–45.41 mm for leaf length, 0.88–3.63 g for fruit weight, and 0.17–0.84 g for stone weight in *Z. spina-christi*. The wide range obtained for leaf and fruit-related characters in the present research was different from previous studies due to differences in the number of samples, environmental conditions, and genetic aspects.

In *Z. nummularia*, CV ranged from 9.90 (in fruit width) to 35.91% (in petiole length). Also, the range of studied traits in this species was as follows: leaf length as 16.45–34.40 mm, leaf width as 9.80–23.70 mm, petiole length as 2.45–6.50 mm, fruit length as 10.32–15.40 mm, fruit width as 12.22–16.30 mm, fruit weight as 0.93–2.02 g, stone length as 7.32–10.80 mm, stone width as 7.30–9.62 mm, and stone weight as 0.31–0.81 g (Table 1). Norouzi et al. [20] reported a range of 16.40–35.90 mm for leaf length, 0.98–2.10 g for fruit weight, and 0.29–0.88 g for stone weight in *Z. nummularia*.

**Fig. 2:**
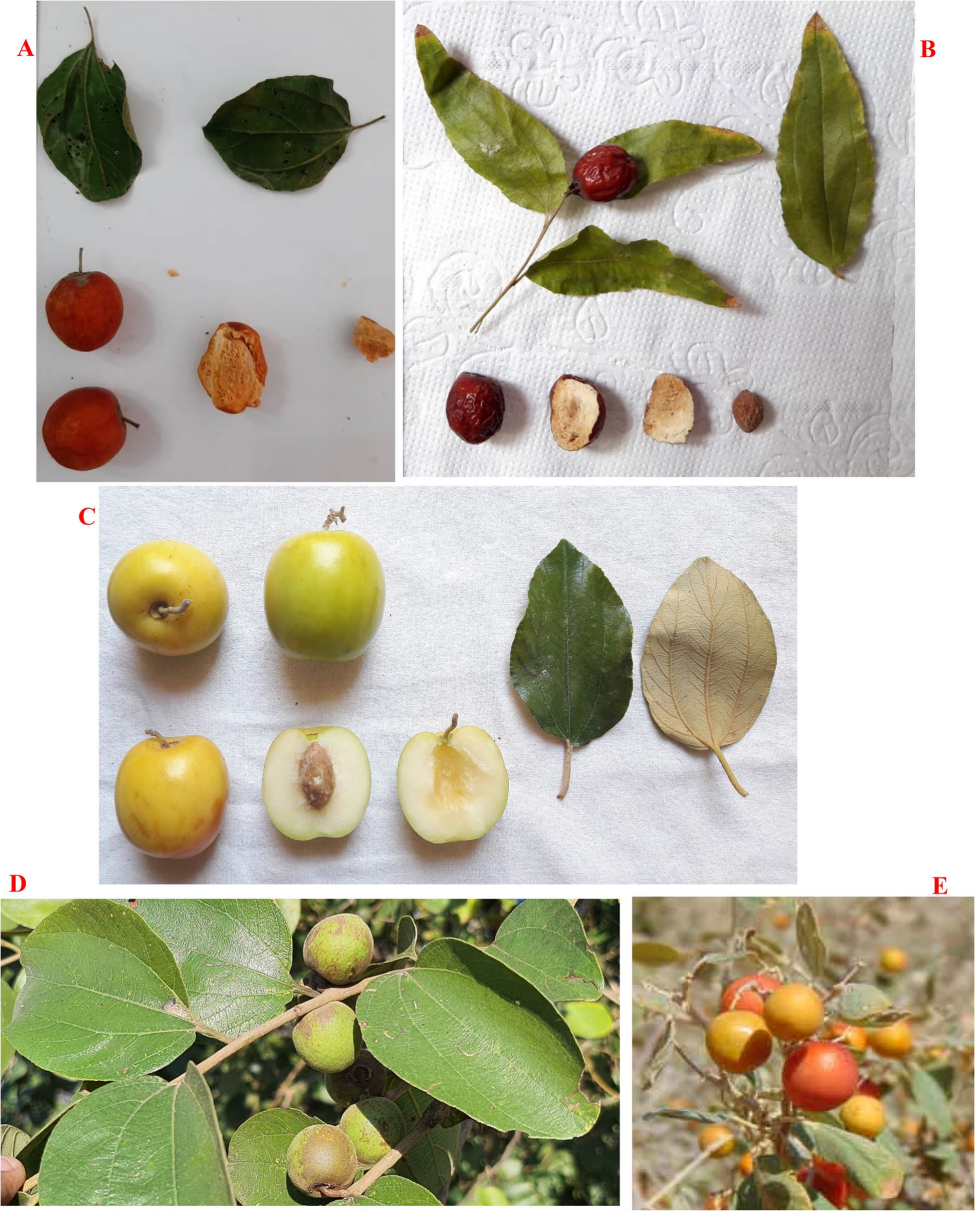
Leaves and fruits of the studied species of the *Ziziphus* genus, including (**A**) *Z. spina-christi*, **B** *Z. jujuba*, **C** *Z. mauritiana*, **D** *Z. xylopyrus*, and **E** *Z. nummularia*

In *Z. xylopyrus*, CV ranged from 8.58 (in leaf width) to 41.02% (in fruit weight). Also, the range of studied traits in this species was as follows: leaf length as 23.02–34.40 mm, leaf width as 17.02-23.00 mm, petiole length as 4.10-7.00 mm, fruit length as 11.45–16.33 mm, fruit width as 12.22–18.65 mm, fruit weight as 0.91–3.02 g, stone length as 7.33–10.70 mm, stone width as 7.02–9.44 mm, and stone weight as 0.35–0.82 g (Table 1). Norouzi et al. [20] reported a range of 23.10–40.70 mm for leaf length, 1.55–3.14 g for fruit weight, and 0.22–0.90 g for stone weight in *Z. xylopyrus*. The pictures of leaves and fruits of the studied species of the *Ziziphus* genus are shown in Fig. 2.

The combined data of all the studied species was used for further analysis. All the measured traits showed significant and positive correlations with each other (Table 2) and corresponded with previous findings in different species of the *Ziziphus* genus [17–23]. Correlation coefficients provide information about important traits in the evaluation of genotypes [24]. These coefficients can be used to describe other variables and considered to describe genotypes [25].

**Table 2:** Simple correlations among the morphological variables utilized in the studied accessions belonging to five species of the *Ziziphus* genus

Trait	Leaf length	Leaf width	Petiole length	Fruit length	Fruit width	Fruit weight	Stone length	Stone width	Stone weight
Leaf length	1								
Leaf width	0.94**	1							
Petiole length	0.88**	0.95**	1						
Fruit length	0.89**	0.90**	0.91**	1					
Fruit width	0.90**	0.91**	0.92**	0.98**	1				
Fruit weight	0.74**	0.80**	0.87**	0.92**	0.89**	1			
Stone length	0.91**	0.90**	0.87**	0.98**	0.96**	0.86**	1		
Stone width	0.82**	0.82**	0.75**	0.83**	0.87**	0.62**	0.88**	1	
Stone weight	0.53**	0.63**	0.73**	0.78**	0.74**	0.89**	0.72**	0.50**	1

**Correlation is significant at *p* ≤ 0.01 level

Multiple regression analysis (MRA) results (Table 3) showed that fruit length, stone width, stone weight, stone length, and fruit width have significant effects on fruit weight, and thus their fluctuations have a significant effect on increasing or decreasing fruit weight. Therefore, breeders should pay attention to the above traits to improve the fruit weight of commercial species of *Ziziphus*. Significant effects of the above characters on fruit weight have been detected using MRA in different fruits [26–28].

**Table 3:** The traits associated with fruit weight in the studied accessions belonging to five species of the *Ziziphus* genus as revealed using MRA and coefficients

Dependent trait	Independent trait	*r*	*r*^2^	*β*	*t*	*p*
Fruit weight	Fruit length	0.92 a	0.86	1.09	5.98	0.00
	Stone width	0.96 b	0.94	-0.33	-6.18	0.00
	Stone weight	0.97 c	0.96	0.28	8.46	0.00
	Stone length	0.98 d	0.97	-0.44	-3.19	0.00
	Fruit width	0.99 e	0.97	0.32	2.83	0.01

**Fig. 3:**
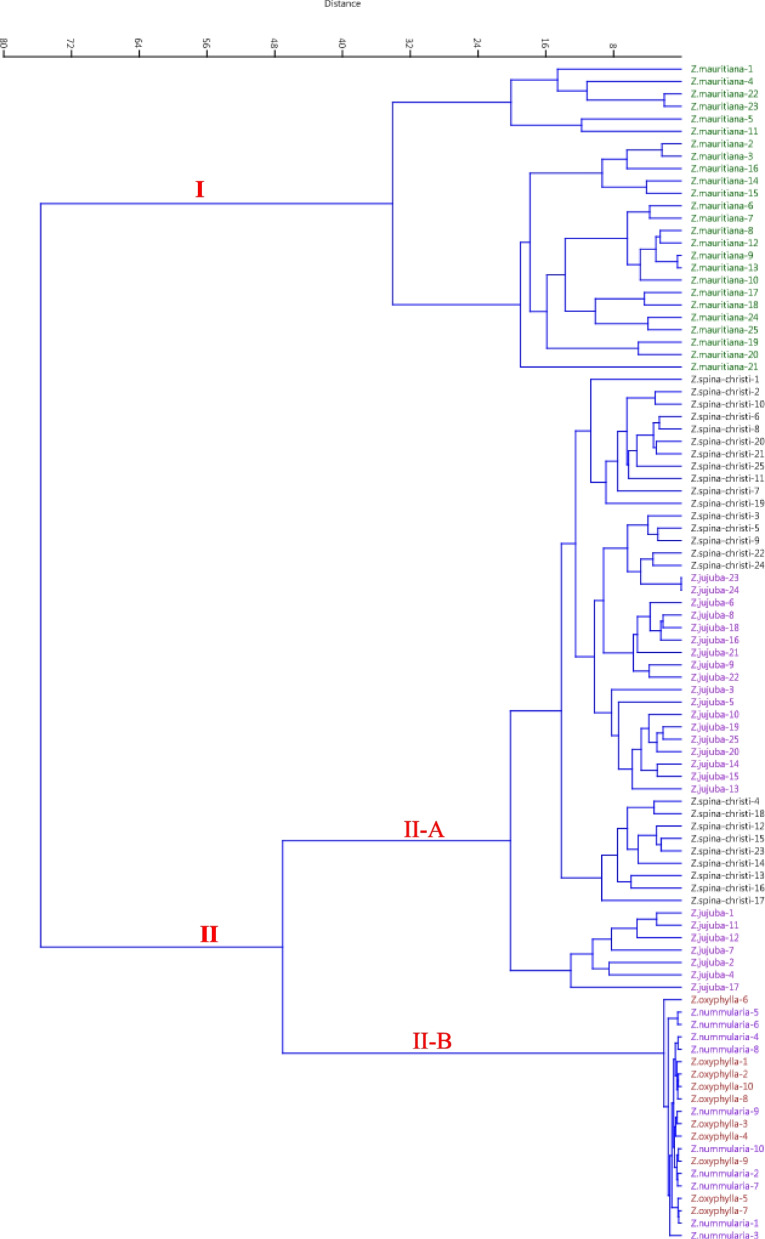
Ward cluster analysis of the studied accessions belonging to five species of the *Ziziphus* genus based on morphological traits using Euclidean distances

Cluster analysis grouped the accessions into two main clusters (Fig. 3). The first cluster (I) included all the accessions of *Z. mauritiana*, while the second cluster (II) included the accessions of the rest species forming two sub-clusters. Sub-cluster (II-A) included the accessions of *Z. spina-christi* and *Z. jujuba* species, while sub-cluster (II-B) consisted of the accessions of *Z. nummularia* and *Z. oxyphylla* species.

**Fig. 4:**
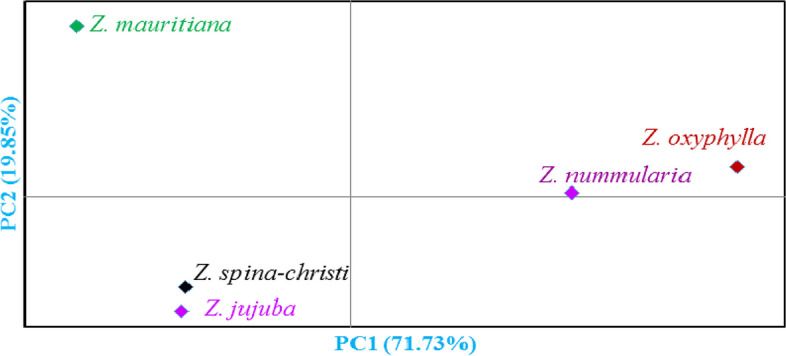
Bi-plot for the studied five species of the *Ziziphus* genus based on the morphological characters

An analysis was performed to determine relationships among the studied species. The five species were placed into three groups (Fig. 4). *Z. nummularia* and *Z. oxyphylla* species were placed into the first group, and the second group included *Z. mauritiana*, while *Z. spina-christi* and *Z. jujuba* species formed the third group.

The genetic diversity of native accessions and their wild relative species is the primary source of many agricultural research programs, especially cross-breeding programs. Therefore, it is necessary to know the characteristics and potential of these valuable resources collected to use them in research programs [22, 23]. With this approach, the accessions studied belonging to the *Ziziphus* genus showed high variability. Higher fruit weight with higher yield capacity is the most important fruit characteristic in breeding programs [21].

The accessions showed considerable variation in terms of the measured traits within and among species studied. The variation among the accessions of the same species is due to cross-pollination, natural hybridization, cross-incompatibility, propagation by seeds, gene flow, and exchange of plant material between the study areas [20]. Also, the dissimilarity between accessions of the species denotes the capability of generating new progenies and producing different associations or segregations of genes, thereby facilitating a partial removal of former linkages or the creation of new ones that can be applied in both classical and modern breeding methods. To generate new progenies in a subsequent generation (with new linkage groups or new population properties), it is a common practice to use distant genotypes [21].

## Conclusions

Phenotypic diversity provides the possibility of selecting high-quality genotypes. Investigation of the morphological characterization of plant species provides the possibility of selecting better accessions for the development of cultivation and leads to more attention of farmers and plant breeders, and those involved in the preservation of plant resources. The studied native accessions belonging to five species of the *Ziziphus* genus can be considered the most important sources of germplasm. The results of the present study can be used in breeding programs and increasing performance in the future. Based on the commercial characters, accessions no. 12, 13, 17, 23, and 24 in *Z. jujuba*, accessions no. 3, 9, 17, 18, 20, 22, and 23 in *Z. mauritiana*, accessions no. 5, 6, 8, 13, 19, 22, and 24 in *Z. spina-christi*, accessions no. 3, 7, and 9 in *Z. nummularia*, and accessions no. 2, 4, 7, and 10 in *Z. oxyphylla* showed the highest fruit weight and thus can be suggested as superior for cultivation or use in breeding programs due to having larger fruits.

## Data Availability

The findings supporting the present study, when reasonable request, are available from the corresponding author.
